# Identification and Characterization of T5-Like Bacteriophages Representing Two Novel Subgroups from Food Products

**DOI:** 10.3389/fmicb.2018.00202

**Published:** 2018-02-13

**Authors:** Domonkos Sváb, Linda Falgenhauer, Manfred Rohde, Judit Szabó, Trinad Chakraborty, István Tóth

**Affiliations:** ^1^Institute for Veterinary Medical Research, Centre for Agricultural Research, Hungarian Academy of Sciences, Budapest, Hungary; ^2^Institute of Medical Microbiology, Justus Liebig University Giessen and German Center for Infection Research (DZIF), Partner Site Giessen-Marburg-Langen, Giessen, Germany; ^3^Central Facility for Microscopy, Helmholtz Centre for Infection Research, HZI, Braunschweig, Germany; ^4^Department of Medical Microbiology, Faculty of Medicine, University of Debrecen, Debrecen, Hungary

**Keywords:** bacteriophages, T5-like phages, phage genomics, Enterobacteriaceae, ESBL *E. coli*, food safety, bio-control

## Abstract

During recent years, interest in the use of bacteriophages as biocontrol agents against foodborne pathogens has increased, particularly for members of the family Enterobacteriaceae, with pathogenic *Escherichia coli, Shigella, and Salmonella* strains among them. Here, we report the isolation and characterisation of 12 novel T5-like bacteriophages from confiscated food samples. All bacterophages effectively lysed *E. coli* K-12 strains and were able to infect pathogenic *E. coli* strains representing enterohaemorrhagic (EHEC), enteropathogenic (EPEC), enterotoxigenic (ETEC), and enteroinvasive (EIEC) pathotypes, *Shigella dysenteriae, S. sonnei* strains, as well as multidrug-resistant (MDR) *E. coli* and multiple strains representing different *Salmonella enterica* serovars. All the bacteriophages exhibited *Siphoviridae* morphology. Whole genome sequencing of the novel T5-like bacteriophages showed that they represent two distinct groups, with the genome-based grouping correlating to the different host spectra. As these bacteriophages are of food origin, their stability and lack of any virulence genes, as well as their broad and mutually complementary host spectrum makes these new T5-like bacteriophages valuable candidates for use as biocontrol agents against foodborne pathogenic enterobacteria.

## Introduction

Several members of the Enterobacteriaceae family are considered significant foodborne pathogens, with enterohemorrhagic *Escherichia coli* (EHEC) and *Shigella* strains capable of causing life-threatening disease even at a very small infectious dose (reviewed in Croxen et al., 2013; Anderson et al., 2016). Many of these infections are treated with antibiotic therapy, but this is threatened by the increasing occurrence of multidrug-resistant (MDR) strains (reviewed in Szmolka and Nagy, 2013). The development of novel antibiotics has significantly slowed down in the past decades, as it is becoming increasingly hard to find agents with new effect mechanisms or targets, and also because of limited economic returns (Hunter, 2012). More recently, attention has turned on the development of “evolution-proof” antibiotics (Bell and MacLean, 2017) and as an alternative answer to this challenge, there is also a renewed interest in the study of bacteriophages capable of lysing pathogenic bacteria.

As biocontrol agents, phages can be applied either as a treatment of ongoing infections (phage therapy) as in the case of *Staphylococcus aureus* strains, or for foodborne pathogens, the treatment of alimentary food as a preventive measure (reviewed by Johnson et al., 2008 and Hagens and Loessner, 2010). There are already several examples of commercially available phage cocktails to be utilized against various foodborne pathogens. There is a phage cocktail aimed against *E. coli* O157 to be applied to food surfaces (Carter et al., 2012), as well as cocktails against *Shigella* (Soffer et al., 2017), *Listeria monocytogenes* and *Salmonella enterica* (reviewed by Abedon, 2017). There have also been clinical trials of phage cocktails against diarrheagenic *E. coli* on human subjects (Sarker et al., 2012, 2016), but these did not yield results regarding the efficacious use of phage cocktails for such infections.

Bacteriophage T5 was one of the first enterobacterial phages to be characterized in-depth in the 1950s, and since then several similar bacteriophages have been reported. Their most unique feature is a two-step DNA transfer during infection (reviewed in Davison, 2015). Later, partly because of the renewed interest of utilizing bacteriophages as antibacterial agents, the bacteriophage T5 genome was fully sequenced (Wang et al., 2005) and new members of this bacteriophage group have also been characterized (Kim and Ryu, 2011; Golomidova et al., 2015). All T5 bacteriophages seem to share a common genomic structure where pre-early, early and late regions can be distinguished depending on the time of transcription during the infectious cycle (reviewed by Davison, 2015). In recent studies, several T5-like bacteriophages were proposed as potential candidates for use against various foodborne pathogens, including *Salmonella enterica* serovar Typhimurium (Kim and Ryu, 2011; Piya et al., 2015) as well as against *E. coli* O157:H7 strains (Niu et al., 2012; Hong et al., 2014), with promising *in vivo* results produced in live sheep with the phage CEV2 (Raya et al., 2011).

In a previous study, we assessed the risk posed by foodborne pathogens present in illegally imported foodstuff in Europe, with a special emphasis on STEC (Nagy et al., 2015). We hypothesized that bacteriophages capable of lysing foodborne pathogenic bacteria should be also capable of surviving and replicating in the same food. Therefore, a potent source for bacteriophage candidates to be applied as biocontrol against foodborne bacteria could be the very same food samples. As pointed out by Abedon (2017), studies aiming toward phage therapy tend to focus on successes on therapeutic application, and neglect in-depth characterisation of phages. Studies on the potential therapeutic application of phages against enterobacterial infections (Sarker et al., 2016) suggest that a preventive approach, i.e., prior eradication of pathogens from food with phages before consumption could be a more succesful application.

In light of these considerations we aimed to isolate bacteriophages from the food samples that are capable of lysing foodborne enteric pathogens to extensively characterize their host spectrum with other phenotypic features, and to characterize their genomes. This in-depth characterisation would serve to determine whether the isolated phages can be considered to serve as biocontrol agents against fooborne pathogenic Enterobacteria.

Using *E. coli* K-12 strains for propagation, we isolated 12 new T5-like lytic bacteriophages from individual food samples, and proceeded with their detailed characterisation. Whole genome sequence analysis revealed that these bacteriophages do not harbor undesirable virulence-related genes. They represent two distinct new genotypes among T5-like bacteriophages and proved to be capable of lysing a wide array of pathogenic *E. coli, Shigella*, and *Salmonella* strains.

## Materials and methods

### Isolation of bacteriophages

Bacteriophages were isolated from food samples confiscated on the Hungarian border by the customs agents. Samples underwent the first steps of the ISO 16654:2001 method for isolating *E. coli* O157. Briefly, 5 g of the food samples were homogenized in 10 volumes of tryptic soy broth (TSB) supplemented with bile salts, and incubated for 24 h at 42°C. Bacterium-free samples of these precultures were spread or spotted onto layered 0.7% soft agar plates containing the *E. coli* K-12 derivative C600 as propagating strain, as described by Strauch et al. (2001). After overnight incubation at 37°C, single plaques were picked and purified by re-propagation on *E. coli* C600 as well as at *E. coli* MG1655 at least three times, until high titer (≥10^11^ PFU/ml) bacteriophage stocks were produced. The list of the isolated bacteriophages and their origin is given in Table 1.

**Table 1:** Origin and subgrouping of new T5-like bacteriophages.

**Phage no**.	**Foodstuff of origin**	**Country of origin**	**Subgroup**
chee24	cattle cheese	Bulgaria	chee24
pork27	row pork meat	Serbia	chee24
pork29	row pork meat	Serbia	chee24
saus47N	pork sausage	Serbia	chee24
saus111K	pork sausage	Ukraine	chee24
poul124	poultry meat	Ukraine	chee24
chee130_1	cattle cheese	Ukraine	chee130_1
saus132	pork sausage	Ukraine	chee130_1
poul149	poultry meat	Ukraine	chee130_1
chee158	cattle cheese	Ukraine	chee130_1
cott162	cattle cottage cheese	Ukraine	chee130_1
saus176 N	pork sausage	Hungary	chee130_1

### Testing host specificity and efficiency of plating

The host specificity of the isolated bacteriophages was investigated by spot assays on the strains listed in Table 2 as described before. In addition to reference strains and strains from our strain collection, extended-spectrum β-lactamase (ESBL)-producing multidrug-resistant (MDR) *E. coli* strains isolated from human clinical samples were investigated. MDR strains were isolated at the University of Debrecen and *Salmonella* strains were kindly provided by László Makrai (University of Veterinary Medicine, Budapest). *Shigella flexneri* M90T was kindly contributed by Zoltán Tigyi (University of Pécs). Enteroinvasive *E. coli* (EIEC) and *Shigella* strains (except for *S. sonnei* 866-F) originated from the Hungarian National Collection of Medical Bacteria (HNCMB), Budapest.

**Table 2:** Host specificity and efficiency of plating of the two subgroups of new T5-like bacteriophages.

**Strain**	**Pathotype/serovar /species**	**Serogroup or serotype**	**Phage chee24**	**Phage chee130_1**	**Strain reference**
II95-36	EIEC	O121	−	−	this study
20	EIEC	O124	−	−	this study
Bra2 26	EIEC	O152	+++	+++	this study
Saigon	EIEC	O164	−	−	this study
E2348/69	EPEC	O127:H6	−	+	Iguchi et al., 2009
536	UPEC	O6:K15:H31	−	−	Hochhut et al., 2006
IHE3034	ExPEC	O18:K1:H7	−	−	Moriel et al., 2010
E250	APEC	O1:K1:H7	−	−	Tóth et al., 2003
T22	atypical	O157:H43	−	−	Tóth et al., 2009
E22	EHEC	O103:H2	++	−	Marchès et al., 2003
Sakai	EHEC	O157:H7	−	−	Hayashi et al., 2001
EDL933	EHEC	O157:H7	−	−	Perna et al., 2001
ICC169	*Citrobacter rodentium*	N/A	−	−	Wiles et al., 2005
20080	*Shigella dysenteriae* 1A	N/A	+++	−	this study
M90T	*Shigella flexner*i	N/A	−	−	this study
20038	*Shigella boydii*	N/A	−	−	this study
866-F	*Shigella sonnei*	N/A	+++	+++	Allué-Guardia et al., 2011
20045	*Shigella sonnei*	N/A	+++	−	this study
1201	*Salmonella* Typhimurium 1	N/A	+++	−	this study
1202	*Salmonella* Infantis	N/A	+	−	this study
1203	*Salmonella* Panama	N/A	+++	−	this study
1199	*Salmonella* Typhi	N/A	−	−	this study
1198	*Salmonella* Gallinarum	N/A	−	−	this study
1200	*Salmonella* Enteritidis	N/A	−	−	this study
5871^*^	ESBL *E. coli*	O15	−	−	this study
18531^**^	ESBL *E. coli*	O73	++	+	this study
29095^**^	ESBL *E. coli*	O90	−	−	this study
H10407	ETEC	O78:H11:K80	+	+	Evans et al., 1973
MG1655	*E. coli* K-12	O16:H48	+++	+++	Blattner et al., 1997

*The range of EOP values relative to the titer of the bacteriophages on Escherichia coli MG1655 is symbolized as follows: +++: between 1 and 10^−3^; ++: between 10^−4^−10^−6^; +: 10^−7^ and below; −: no lysis*.

* *Resitance to ESBL, Ciprofloxacin, Gentamicin, Tetracycline*.

** *Resistance to ESBL, Ciprofloxacin, Gentamicin, Sulphamethoxazole/Trimethoprim*.

The ESBL resistant *E. coli* strains were isolated from human extraintestinal infections, the *Shigella flexneri* strain was isolated from human feces and the *Salmonella* strains were isolated from food or from animals. These isolates were identified biochemically and were serotyped using O specific immunsera (data not shown).

Efficiency of plating (EOP) was determined by applying serial dilutions of bacteriophage suspensions in spot assays. The ratio of bacteriophage titer on each strain to the titer measured on *E. coli* MG1655 was considered the EOP of the bacteriophage on the given strain.

### One step growth experiments

One step growth experiment to determine burst size was conducted on the representative bacteriophages chee24 and chee130_1 on *E. coli* MG1655, according to the description of E. Bassiri^1^ with some modifications. Briefly, 5 × 10^8^ bacteria were mixed with 5 × 10^6^ bacteriophages in Luria broth (LB), setting the multiplicity of infection (MOI) to 0.01. The mixture was incubated for 10 min at room temperature for initial adsorption, then diluted 10^4^-fold, and in a total volume of 10 ml incubated at 37°C for 1 h. Hundred microliter aliquots were taken every 5 min and plated on layered soft agar for counting. Experiments were run at three independent times in two parallels each. Burst size was determined as a ratio of average bacteriophage count of the baseline and the average bacteriophage count after the burst.

### Heat stability tests

Heat stability of the bacteriophages was tested as follows: 1 ml stocks containing a 100-fold dilution of a bacteriophage stock in LB were incubated for 1 h at 25°C, 37°C, 42°C, and 80°C. After incubation, the titer of the treated bacteriophage stocks was determined with spot assay on *E. coli* MG1655. Experiments were performed in two parallels on each temperature.

### pH tolerance tests

Stability of the bacteriophages under different pH values was tested by incubating 100-fold dilutions of the bacteriophages for 1 h at 37°C in 1 ml LB pH-adjusted to 3, 5, 7, and 9 with HCl and NaOH solutions. After incubation, the titer of the treated bacteriophage stocks was determined with spot assay on *E. coli* MG1655. Experiments were performed in two parallels on each pH value.

### Determination of bacteriophage morphology

Bacteriophages were investigated by transmission electron microscopy (TEM). Briefly, drops of a high titer bacteriophage suspensions were placed on parafilm, absorbed onto carbon film, washed in TE buffer (10 mM TRIS, 1 mM EDTA, pH 6.9) and negatively-stained with 2% aqueous uranyl acetate, pH 5.0. Carbon film was collected with 300 mesh copper grids and access negative-stain was removed with filter paper and subsequentely air-dried. Samples were examined in a TEM 910 transmission electron microscope (Carl Zeiss, Oberkochen) at an acceleration voltage of 80 kV. Images were recorded digitally at calibrated magnifications with a Slow-Scan CCD-Camera (ProScan, 1024 × 1024, Scheuring, Germany) with ITEM-Software (Olympus Soft Imaging Solutions, Münster, Germany). Contrast and brightness were adjusted with Adobe Photoshop CS3.

### Bacteriophage DNA isolation

Bacteriophage DNA was isolated from bacteriophage stocks with a concentration of at least 10^11^ PFU/ml. The phenol-chloroform method described by Sambrook et al. (1987) was used for isolation with the modifications outlined by Tóth et al. (2016). Briefly, to remove any non-bacteriophage related DNA and RNA from the 700 μl bacteriophage suspension, the sample was treated with amplification grade DNase I and RNase A (Sigma Aldrich) in 10 μg/ml final concentration for 30 min at 37°C. For bacteriophage lysis, proteinase K (Sigma Aldrich) was added in 6.6 μg/ml final concentration to the lysis buffer (50 mM Tris–HCl (pH 8.0), 10 mM EDTA, 0.5% SDS) and incubated at 65°C for 30 min. The suspension was cooled to room temperature and mixed with 750 μl of a 1:1 mixture of equilibrated phenol and chloroform. After 5 min incubation at room temperature, proteins were removed by centrifugation at 10,000 × g for 5 min. The aqueous phase was transferred to a clean Eppendorf tube and bacteriophage DNA was precipitated after adding 0.1 volume of 3 M potassium acetate, pH 5.5 with 0.7 volume of isopropanol at ice for 20 min. DNA was collected by centrifugation at 13,000 × g at 10 min, washed with 600 μ1 70% ethanol and dissolved in 30 μl 10 mM Tris–HCl, 1 mM EDTA, pH 8.0 buffer.

### Bacteriophage genome sequencing and analysis

Genomic DNA sequencing libraries were prepared using the Nextera XT kit (Illumina, Eindhoven, NL). Sequencing was performed using Nextseq Mid-output reagent kit v2 (2 × 150 bp) on an Illumina NextSeq 500. Assembly was performed with SPAdes (Bankevich et al., 2012). The genome was annotated using the RAST server (Overbeek et al., 2014). The genes orf5c (T5.035) and the bacteriophage DNA polymerase (T5.122) were used for phylogenetic analysis. The DNA sequences of these two genes from all bacteriophages determined in this study and of T5 reference genomes (Supplementary Table 1) were aligned using Clustal Omega (Sievers et al., 2011). The phylogenetic tree was visualized using MEGA5.2 (Hall, 2013). For whole-bacteriophage sequence phylogeny, the program VICTOR (Meier-Kolthoff and Goeker, 2017) was used with default settings and all T5 reference genomes (Supplementary Table 1). Determination of sequence homologies and the identification of SNPs were conducted by using the BLAST tools available at the NCBI website. Genome alignments were visualized with Easyfig (Sullivan et al., 2011) and modified using Inkscape. The left and right repeats were defined using a pile-up analysis of raw sequencing reads after mapping with CLC workbench 9.0 (Qiagen, Hilden, Germany). The repeats were defined as >130% of average coverage at the particular site (30% more coverage than average coverage).

### Sequence accession numbers

The whole genome sequences of all bacteriophages determined in the current study were deposited in GenBank database under accession nos. MF431730- MF431741.

## Results

### Isolation of bacteriophages

By using *E. coli* K-12 derivative strains MG1655 and C600 as indicator and propagating strain, we isolated 12 new bacteriophages from different food samples. The origin, as well as the subgrouping of the bacteriophages are summarized in Table 1. The bacteriophages were isolated from either meat, meat products (e.g., sausages) or cheese.

### Morphology

**Figure 1:**
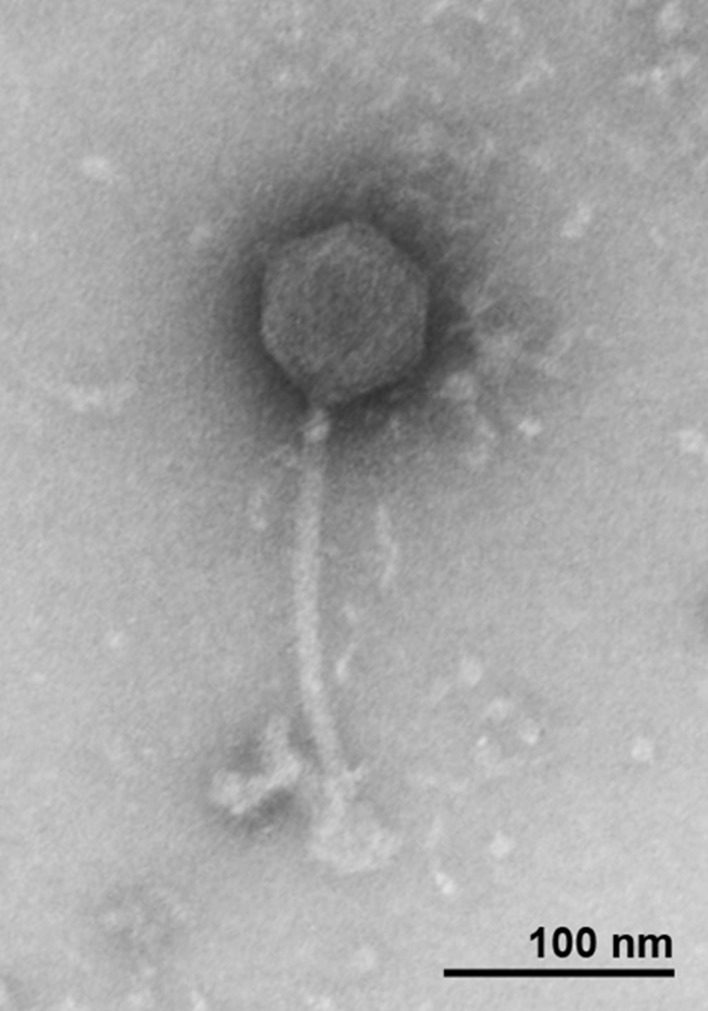
Transmission electron micrograph of bacteriophage 24 showing Siphoviridae morphology.

All the new isolated bacteriophages showed Siphoviridae morphology, with an average head length of ~85 nm, head width of ~75 nm and a flexible, non-contractile tail with a length of ~200 nm. As a representative, the morphology of T5-like bacteriophage chee24 is shown (Figure 1).

### Host spectrum, efficiency of plating, burst size, and stability

To assess the host specificity of the bacteriophages, we tested their lytic capacity on a large variety of enterobacterial pathogenic and ESBL multidrug-resistant clinical strains listed in Table 2. We found that the novel T5-like bacteriophages showed two distinct lysis patterns, which we denote by a representative bacteriophage of each: bacteriophage chee24 (group chee24) and bacteriophage chee130_1 (group chee130_1). The grouping of the bacteriophages is indicated in Table 1. EOP values were determined by spot assay for these two bacteriophages. The lysis spectrum and EOP values are given in Table 2.

Apart from K-12 derivative strains, several *E. coli, Shigella*, and *Salmonella* strains representing different pathotypes and serovars proved to be susceptible to the bacteriophages. While bacteriophage chee130_1 lysed enteropathogenic *E. coli* (EPEC), EIEC, and *Shigella sonnei* strains, bacteriophage 24 induced lysis on *Salmonella* serovars, EHEC O103:H2, and *Shigella dysenteriae* strains. Both bacteriophages showed lysis on enterotoxigenic *E. coli* (ETEC) type strain H10407. It has to be noted however, that in each case EOP values were orders of magnitude lower when compared to K-12 strains, and on some wild type strains the plaque morphology was opaque, unlike the clear plaques of ~2 mm in diameter, observed with the strain MG1655.

**Figure 2:**
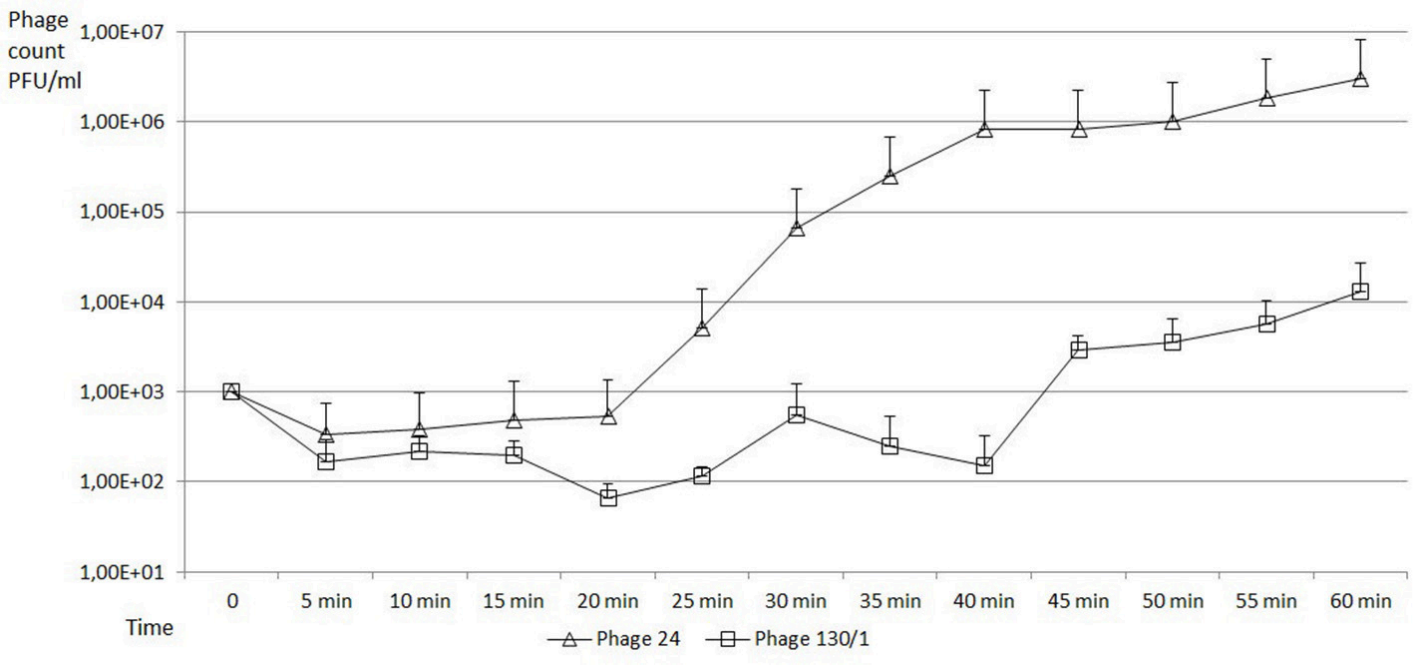
One step growth curves of new T5-like bacteriophages on *E. coli* MG1655 strain. PFU values of bacteriophage chee24 are marked with triangles, those of bacteriophage chee130_1 are marked with squares. The error bars indicate standard deviations from the results of the three independent triplicate experiments.

Burst size was determined with one step growth experiments on *E. coli* MG1655 (Figure 2). The burst sizes of bacteriophage chee24 and chee130_1 proved to be different, in the case of the former, it was around 1,000 PFU/cell, while in the case of chee130_1, the burst size was around 44 PFU/cell. In both cases the burst occurred ~45 min after the end of the initial absorption.

Heat stability experiments showed that incubation of 10^10^ PFU bacteriophage stocks at 25°C, 37°C and 42°C for 1 h did not significantly affect the EOP of the bacteriophages (data not shown). Incubation at 80°C, however, caused 10^4^-fold average decrease in the bacteriophage titer.

A pH value of 3 completely deactivated the bacteriophages, as no plaques were observable even in the spots of the concentrated suspensions. On the other hand, no significant decrease in titer could be observed at pH values 5, 7, and 9 after 1 h incubation (data not shown).

### Whole genome sequence analysis

To examine the genomic structures and the potential diversity in the genomes, all the 12 newly isolated bacteriophage genome sequences were determined and detailed genomic comparison was conducted. Genome sequencing of the bacteriophages showed that all the bacteriophages have a linear double-stranded DNA genome, and belong to T5-like bacteriophages. The two subgroups with different host specificity also have minor genetic differences. The genome of the “group chee24” bacteriophages is 120,618 (bacteriophage pork27), 120,620 (bacteriophage saus111K), 120,622 (bacteriophage chee24, pork29 and saus47N) or 120,629 bp (bacteriophage poul124) long, and contains 165 (saus47N, saus111K, poul124), 166 (chee24, pork29) or 168 (pork27) protein-encoding CDS, as well as 18 tRNA genes, respectively. Its GC content is 39.3%. On the other hand, the genome size of the ‘group chee130_1’ bacteriophages is 121,986 bp, with 164 (saus132, poul149), 165 (cott162) or 167 (chee130_1, chee158, saus176N) protein-encoding CDS, 20 tRNA genes, and a GC content of 39.7%. These groups were congruent with the different host spectrum observable within the bacteriophages, indicating that the chee24-like and chee130_1-like bacteriophages form distinct genetic groups. The list of ORFs compared to each other and to the original T5 bacteriophage (GenBank AY543070.1) are shown in Supplementary Tables 2, 3. Members of both subgroups showed the characteristic functional regions in their genomes, which is a common feature of T5-like phages, having pre-early, early and late genomic regions named after the time of their transcription, as well as a terminal repeat region on the 5′ and 3′ end, which is 9,968 bp long in chee24-like phages, and 8,717 bp in group chee130_1 phages. For group chee24 bacteriophages, the bacteriophage pork27 differed by 1 SNP in the terminal repeat, while for group chee130_1 phages, all terminal repeats of other than chee130_1 differed from those present in the prototype, with saus132, chee158, cott162 and saus176N having 3 SNPs, and poul149 harboring 4 SNPs.

At the nucleotide level, the newly identified T5-like bacteriophages not only have the same genome size within the two subgroups—group chee24 and group chee130_1-, but when compared to the “type bacteriophage” of their groups as reference bacteriophage chee24 or chee130_1, respectively, they differ in only 2–5 nucleotide positions. It has to be noted that in the case of bacteriophage pork27 and saus47N, one SNP causes a premature stop codon in bacteriophage protein genes of unknown function. In the case of bacteriophages saus132, poul149 and chee158 there are premature stop codons in a bacteriophage tail fiber gene. A summary of these genomic locations is given in Table 3.

**Table 3:** Single nucleotide polymorphisms shown by T5-like bacteriophages of the chee24-like subgroup (A) and the chee130_1-like subgroup (B) when compared to the type bacteriophage of each subgroup.

**A**									
**Position in phage chee24**	**ORF**	**Predicted function**	**chee24**	**pork27**	**pork29**	**saus47N**	saus111K^*^	poul124^**^	**Amino acid change**
24634	42	Phage protein	C	**A**	**A**	C	**A**	**A**	V65L
57794	101	Phage protein	G	G	G	**A**	G	G	W22^*^
84550	129	Phage tail fibers	C	C	C	**T**	**T**	**T**	G700D
84602	129	Phage tail fibers	T	**G**	**G**	**G**	**G**	**G**	T683P
86896	130	Phage protein	C	**A**	C	C	C	C	E59^*^
**B**									
**Position in phage chee130_1**	**ORF**	**Predicted function**	**chee130_1**	**saus132**	**poul149**	**chee158**	**cott162**	**saus176N**	**Amino acid change**
595	2	Phage protein	G	**A**	**A**	G	G	G	synonymous
1774	4	Probable A1 protein	C	C	C	**G**	C	C	G493A
1919	4	Probable A1 protein	G	G	G	G	**A**	G	L445F
2501	4	Probable A1 protein	A	A	**G**	A	A	A	synonymous
2707	4	Probable A1 protein	G	G	G	G	G	**A**	R182W
3301		intergenic region	A	**C**	**C**	**C**	**C**	**C**	N/A
3303		intergenic region	T	**A**	**A**	**A**	**A**	**A**	N/A
50473		intergenic region	T	**C**	**C**	T	T	T	N/A
86944	133	Phage tail fibers	G	G	G	**A**	G	G	Q210^*^
86950	133	Phage tail fibers	C	C	C	C	**T**	C	A212T
87643	133	Phage tail fibers	G	**A**	**A**	G	G	G	Q443^*^

* *In position 101429-101430, AA is deleted*.

** *In position 101429-101430, AA is deleted. At the beginning, ACT is inserted, and at the end, CGTG is inserted*.

*Letters in bold refer to nucleotides different from those found in phages chee24 and chee130_1 in the same positions, respectively*.

None of the new characterized bacteriophage genomes contained any known virulence, toxin, antibiotic resistance-encoding and virulence regulator genes.

### Phylogenetic analysis

**Figure 3:**
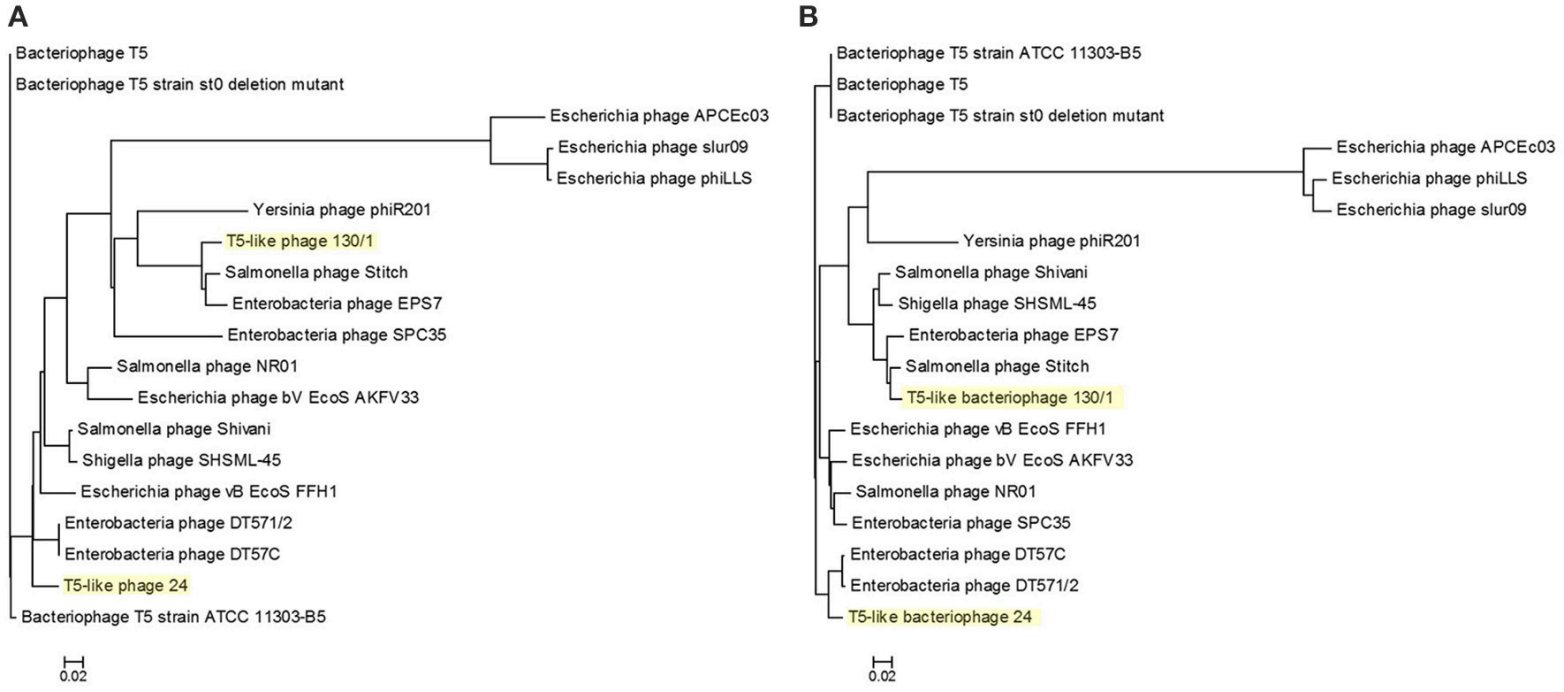
Phylogenetic tree of T5-like bacteriophages based on the Clustal Omega alignment of the genes orf5c (T5.035, **A**) and the phage DNA polymerase (T5.122, **B**) of bacteriophage T5 (AY692264.1). The scale represents homology %. The list of included T5-like phage genomes with their GenBank accession numbers is given in Supplementary Table 1.

To reveal the phylogenetic position of bacteriophages chee24 and chee130_1 among T5-like phages, several comparisons were conducted. Comparison based on the genes orf5c (T5.035) and the bacteriophage DNA polymerase (T5.122) of bacteriophage T5 (AY692264.1) showed that while bacteriophage 24 proved to be the closest relative of *E. coli* bacteriophages DT57C and DT571/2, bacteriophage chee130_1 are closely related to the *Salmonella* bacteriophages Stitch and SPC35 (Figure 3).

**Figure 4:**
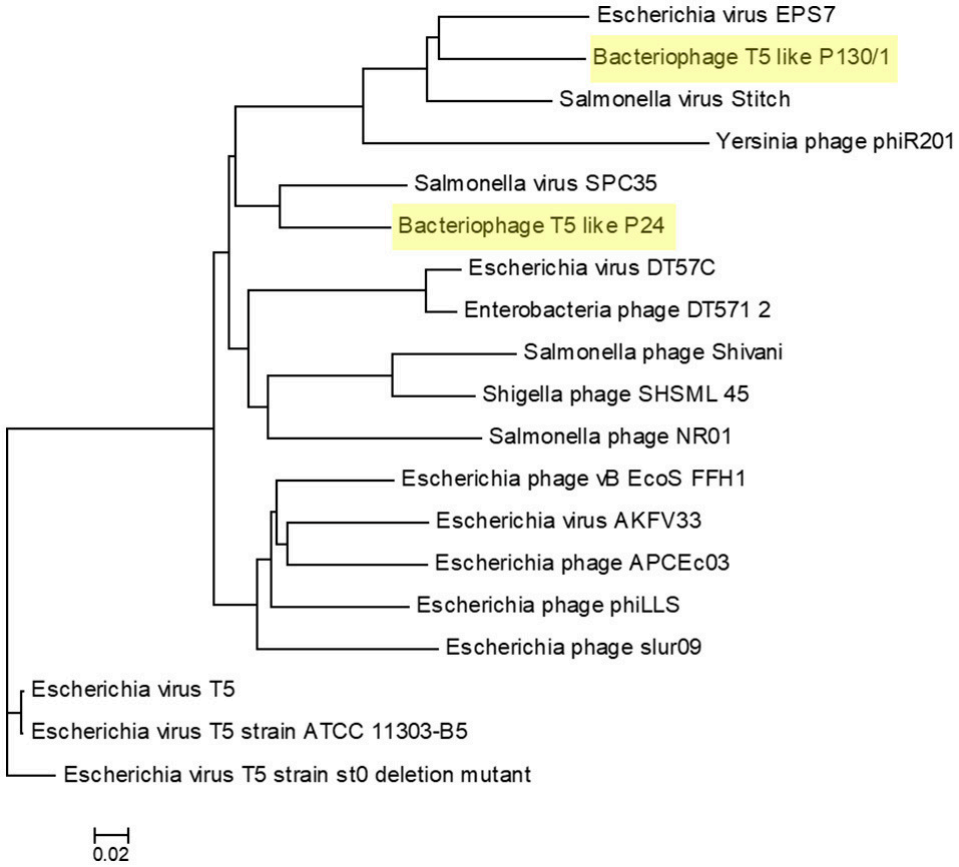
Phylogenetic tree of T5-like bacteriophages made with the whole genome-based VICTOR analysis. The scale represents homology %. The list of included T5-like phage genomes with their GenBank accession numbers is given in Supplementary Table 1.

**Figure 5:**
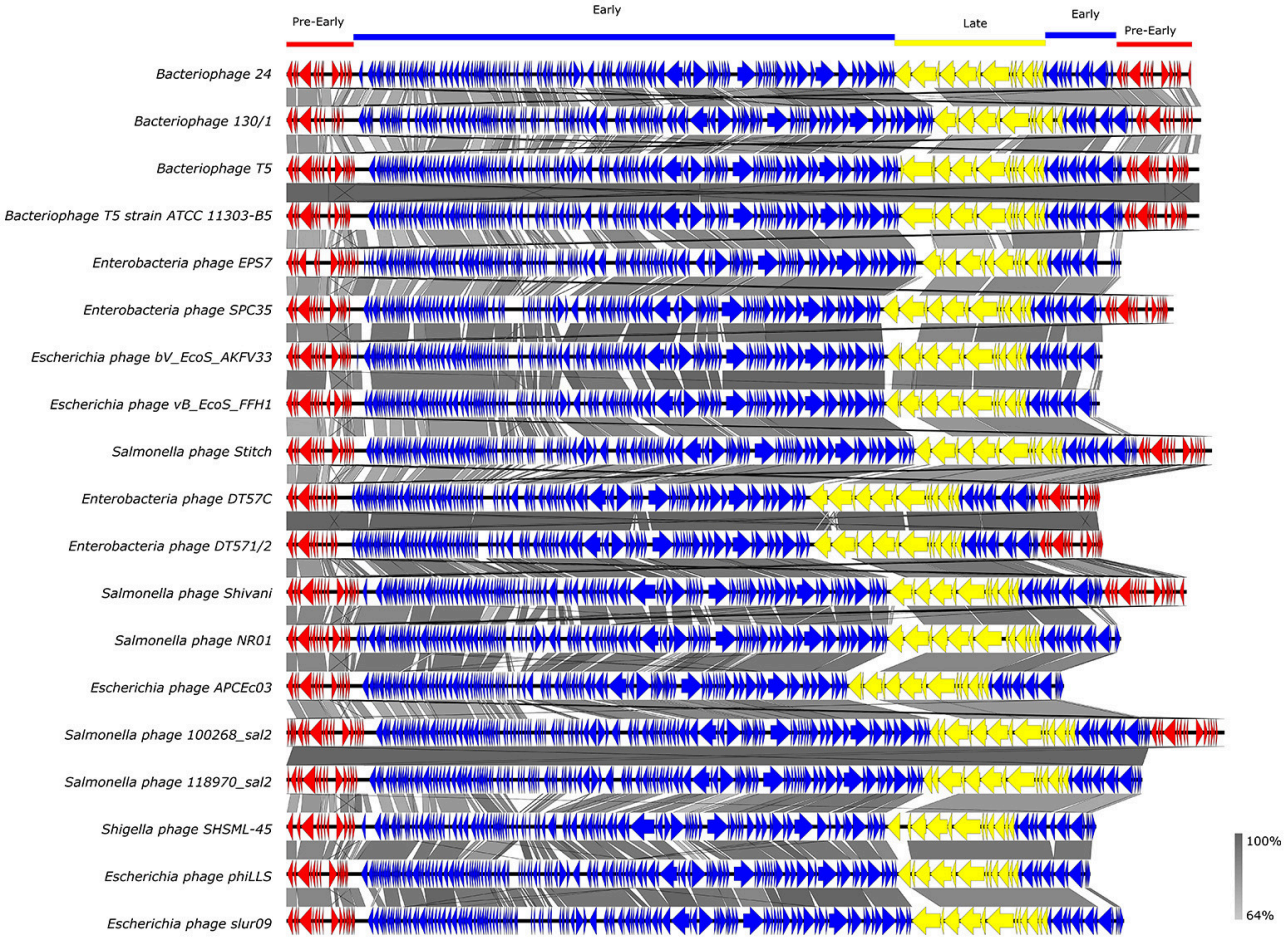
Alignment of T5-like bacteriophage whole genomes. GenBank accession numbers of the phages are given in Supplementary Table 1.Arrows with different colors represent ORFs associated with the genomic regions indicated at the top. The gray bar in the lower right corner shows the identity percentage associated with the color of the bars connecting homologous regions.

Whole-genome based phylogeny of all T5-like bacteriophages clearly indicated that the phages isolated in the current study are members of the T5-like bacteriophages (Figure 4). The topology of this tree is entirely different from the one based on the single genes, putting the two representative phages next to the phage genomes that also proved closest homologs with the BLAST analysis. An alignment of T5-like genomes with mutual homologies including representatives of the two subgroups is presented in Figure 5, showing the 5′ end of the late region as the most variable, which contains mainly tail fiber genes.

Comparing the genomes of bacteriophage chee24 and chee130_1at the nucleotide-level, regions showing low homology to each other were the tail fiber region and an open reading frame encoding a bacteriophage-associated receptor binding protein gene. All these features seem to contribute to the differences observed in the host specificity of the two bacteriophage groups. At nucleotide level, the closest homolog to bacteriophage chee24 is bacteriophage SPC35 (GenBank no. HQ406778.). In addition, two hypothetical proteins, a thymidylate synthase gene, and two subunits of a HNH endonuclease, a receptor binding protein (CDS ADW80123.1) of SPC35 is also absent in chee24, suggesting different host specificity. Bacteriophage EPS7 (GenBank no. CP000917) is the closest homolog to bacteriophage chee130_1 of the previously sequenced T5-like bacteriophages, and carries homologs to all receptor-associated proteins, albeit the similarity is not 100% in each case. Furthermore, chee130_1 lacks a few hypothetical proteins and AGC_0146 putative tail protein as well. Genes showing low homology and those that are absent from bacteriophages of either the chee24 or chee130_1subgroup are listed in Table 4.

**Table 4:** List of genes and regions showing low homology or missing in chee24-like (A) and chee130_1-like (B) bacteriophages when compared to their closest homolog bacteriophages.

**A**			
**Phage chee24**		**Phage SPC35**	
**ORF**	**Predicted function**	**CDS**	**Predicted function**
none	intergenic region	ADW79993.1	hypothetical protein
none	intergenic region	ADW79995.1	hypothetical protein
7	phage protein	ADW79996.1	hypothetical protein
7	phage protein	ADW79997.1	hypothetical protein
none	intergenic region	ADW80009.1	hypothetical protein
31	serine-threonine proteine phosphatase	ADW80013.1	serine-threonine proteine phosphatase
41	phage protein	ADW80023.1	hypothetical protein
41	phage protein	ADW80025.1	hypothetical protein
none	intergenic region	ADW80038.1	hypothetical protein
none	intergenic region	ADW80040.1	hypothetical protein
91	thymidilate synthase	ADW80064.1	thymidilate synthase
none	intergenic region	ADW80067.1	putative H-N-H-endonuclease P-TflVIII
99	phage protein	ADW80071.1	hypothetical protein
none	intergenic region	ADW80078.1	hypothetical protein
134	phage tail-length tape measure protein	ADW80108.1	pore-forming tail tip protein
145	phage tail fibers	ADW80118.1	putative tail protein
149	phage-associated receptor-binding protein	ADW80118.1	receptor-binding protein
**B**			
**Phage chee130_1**		**Phage EPS7**	
**ORF**	**Predicted function**	**CDS**	**Predicted function**
9	phage protein	ACB97450.1	hypothetical protein
none	intergenic region	ACB97451.1	hypothetical protein
none	intergenic region	ACB97452.1	hypothetical protein
none	intergenic region	ACB97458.1	hypothetical protein
none	intergenic region	ACB97467.1	hypothetical protein
26	phage protein	ACB97475.1	hypothetical protein
28	phosphoesterase	ACB97478.1	putative serine/threonine phosphatase
29	phage protein	ACB97479.1	hypothetical protein
30	serine/threonine phosphatase	ACB97480.1	hypothetical protein
31	phage associated homing endonuclease	ACB97482.1	hypothetical protein
none	intergenic region	ACB97510.1	hypothetical protein
none	intergenic region	ACB97513.1	rho-associated, coiled-coil containing protein kinase 2
60	phage protein	ACB97515.1	hypothetical protein
61	hypothetical protein	ACB97516.1	hypothetical protein
none	intergenic region	ACB97518.1	hypothetical protein
65	phage protein	ACB97519.1	hypothetical protein
101	unknown	ACB97556.1	hypothetical protein
105	NAD-dependent protein deacetylase of SIR2 family	ACB97560.1	putative Sir2-like protein
109	hypothetical protein	ACB97569.1	hypothetical protein
134	phage tail fibers	ACB97589.1	putative phage tail protein
139	phage tail-length tape measure protein	ACB97594.1	pore-forming tail tip protein
139	phage tail-length tape measure protein	ACB97595.1	pore-forming tail tip protein pb2
none	intergenic region	ACB97613.1	hypothetical protein

*In the case of chee24-like subgroup the reference bacteriophage is SPC35 (GenBank HQ406778), in the case of chee130_1-like bacteriophages it is EPS7 (GenBank CP000917). Corresponding, albeit in some cases non-homologous ORFs or intergenic regions are displayed in the same row*.

## Discussion

Using *E. coli* K-12 C600 strain as the host bacterium, we isolated and characterized 12 novel T5-like bacteriophages from independent food samples of various animal origin from around the world These novel bacteriophages represented two subgroups based on their host specificity. While the most effective host of the bacteriophages were K-12 derivative *E. coli* strains, several strains representing important intestinal pathotypes of *E. coli, Shigella*, and *Salmonella* also proved susceptible to the bacteriophages. The Siphoviridae morphology and the genomic characteristics including the genome size, the genome architecture, the low GC content and the sequence homologies showed that the bacteriophages are T5-like bacteriophages.

An important feature of these newly found phages is that they were isolated from food samples, unlike the previously isolated T5-like bacteriophages, which originated from sewage and fecal sources (Kim and Ryu, 2011; Golomidova et al., 2015). There is also one case where a T5-like phage was isolated and characterized from a commercially available bacteriophage cocktail designed against enteric pathogens (Piya et al., 2015). Our results indicate that food can be a rich source of bacteriophages effective against pathogenic bacteria potentially residing in the same environment. These phages maintain their infective capacity in this environment suggesting that foodborne phages are promising biocontrol candidates against foodborne bacteria.

Our results also show the huge diversity of bacteriophages, even from well-characterized groups such as T5-like bacteriophages, as both the chee24 and chee130_1 subgroups are different from the closest related bacteriophages. Our data indicate that the most crucial differences are within genes encoding receptor-binding proteins (Table 4, Figure 5). The closest colinear relative of bacteriophage chee24 is SPC35, which was reported to lyse *Salmonella* Typhimurium and Enteritidis strains, as well as a large set of K-12 derivative and commensal *E. coli* (Kim and Ryu, 2011). However, the host spectrum of group chee24 phages is particularly broad, and includes a strain representing one of the so-called “big six” serotypes of EHEC (O103:H2), as well as an ESBL *E. coli* strain of serogroup O73 isolated from an extraintestinal infection. The differences in ORFs 145 and 149, annotated as “phage tail fibers” and “receptor binding protein” could account for the discrepancy.

We wanted to test the efficiency of the new T5-like phages on target strains, and the one step growth on *E. coli* MG1655 yielded burst sizes comparable to those of the T5-like bacteriophage phiLLS (Amarillas et al., 2017).

The closest homolog of the chee130_1 subgroup, EPS7 is able to lyse the same *Salmonella* serovars and seven unnamed *E. coli* strains (Hong et al., 2008), however, phage chee130_1 did not lyse *Salmonella* strains. On the other hand, phage chee130_1 was able to propagate on *Shigella sonnei*, EIEC and EPEC strains. All of these differences in host range underline the potential role that genes showing low homology to closely related bacteriophages (Table 4) might have importance in host specificity. In the case of chee130_1, ORF134 that is annotated as “phage tail fibers” has very low homology to the corresponding gene in EPS7 and seems to support this notion. It is interesting to note however, that in ORF133, another gene annotated as phage tail fiber, there are premature stop codons in the case of bacteriophages saus132, poul149 and chee158. These mutations nevertheless did not seem to have any effect on the bacterial host specificity of these bacteriophages when compared to other members of their subgroup (data not shown).

Lee et al. (2016) suggested that besides bacteriophage cocktails, a solution for simultaneous biocontrol of several foodborne pathogens could be the cloning of several receptor binding genes into a single bacteriophage. For either purpose, obtaining knowledge about new bacteriophages capable of infecting these pathogens and assessing their host spectrum is essential.

Besides the above-cited studies of promising candidate phages to be used as biocontrol agents against pathogenic bacteria, there are already several commercially available phage cocktails aimed at various foodborne enterobacterial pathogens (reviewed by Abedon, 2017). However, according to our knowledge except for ShigaShield, which is aimed against *Shigellae* (Soffer et al., 2017), and the Microgen ColiProteus aimed against *E. coli* and *Proteus* (McCallin et al., 2013), none of the component phages have been sequenced. In order to assess the applicability of a phage either for biocontrol of for therapy, extensive knowledge of its genome is essential, to determine whether the phage carries “undesirable” genes. In-depth knowledge of genes determining host specificity or those contributing to phage stability in different environments are also valuable information when selecting bacteriophages for a given therapeutic or biocontrol purpose.

As it has been noted by Brüssow (2012), the development of an actual phage therapy treatment can take years from phage isolation to performing clinical trials. This process includes for exapmle the purification (Bourdin et al., 2014b), as well as characterisation of host specificity (Bourdin et al., 2014a) and testing the biological safety of the phages either in animals (Raya et al., 2011) or human subjects (Sarker et al., 2012, 2016). An extensive genetic database of phages isolated from different sources, representing different genuses and host spectra could speed up the process of selecting phages for clinical trials.

In summary, we identified 12 new T5-like bacteriophages from food samples, which represent two new species within this bacteriophage group. Our study so far shows that they fulfill important criteria listed by Hagens and Loessner (2010) for bacteriophages to be used as biocontrol: their genome sequence was determined, they propagate well on a non-pathogenic host, they lack any virulence, virulence regulator, toxin, and antibiotic resistance genes. They also seem to be strictly lytic and were shown to be stable during storage and application temperatures, as well as a range of pH values usual in most of the food.

All the above-mentioned properties, coupled with their food origin, as well as their broad and partially overlapping, supplementary host spectrum makes the new T5-like bacteriophages valuable candidates as effective bio-control agents against foodborne pathogenic enterobacteria.

## Author contributions

IT and TC conceived and elaborated the study. DS, LF, MR, JS, and IT conducted the phenotypic experiments. LF performed sequencing and provided bioinformatical tools. DS, LF, TC, and IT analyzed the results and composed the manuscript. All authors reviewed the manuscript.

### Conflict of interest statement

The authors declare that the research was conducted in the absence of any commercial or financial relationships that could be construed as a potential conflict of interest.

## Footnotes

^1^http://www.sas.upenn.edu/LabManuals/biol275/Table_of_Contents_files/13-PhageGrowth.pdf Accessed on 20th September, 2017.
