# Supplementary material for: Identification and Characterization of T5-Like Bacteriophages Representing Two Novel Subgroups from Food Products
Source: Front Microbiol. 2018 Feb 13;9:202. doi: 10.3389/fmicb.2018.00202 (PMC5816814; doi:10.3389/fmicb.2018.00202)
Supplement: Supplementary file 1 [file Table1.docx]

**Supplementary Table 1: T5-like bacteriophages included in the study**

| Description | Accession number |
| --- | --- |
| Bacteriophage T5 | AY543070.1 |
| Bacteriophage T5 strain ATCC 11303-B5 | AY587007.1 |
| Bacteriophage T5 strain st0 deletion mutant | AY692264.1 |
| Enterobacteria phage EPS7 | CP000917.1 |
| *Yersinia* phage phiR201 | HE956708.2 |
| Enterobacteria phage SPC35 | HQ406778.1 |
| *Escherichia* phage bV_EcoS_AKFV33 | HQ665011.1 |
| *Escherichia* phage vB_EcoS_FFH1 | KJ190157.1 |
| *Salmonella* phage Stitch | KM236244.1 |
| Enterobacteria phage DT57C | KM979354.1 |
| Enterobacteria phage DT571/2 | KM979355.1 |
| *Salmonella* phage Shivani | KP143763.1 |
| *Salmonella* phage NR01 | KR233164.1 |
| *Escherichia* phage APCEc03 | KR422353.1 |
| *Shigella* phage SHSML-45 | KX130863.1 |
| *Escherichia* phage phiLLS | KY677846.1 |
| *Escherichia* phage slur09 genome assembly slu09, chromosome : 1 | LN887948.1 |

**Supplementary Table 2. Comparative list of ORFs in T5-like bacteriophages representing the chee24-like subgroup.**

| **ORF** | **Strand** | **Predicted function** | **Corresponding ORF in T5 or chee130_1** |
| --- | --- | --- | --- |
| 1 | - | degrades 5'-deoxyribonucleotides to their corresponding deoxyribonucleosides | AAS77048 |
| 2 | - | Phage protein | AAS77049 |
| 3 | - | hypothetical protein | none |
| 4 | - | Probable A1 protein | AAS77051 |
| 5 | - | A2 protein | AAS77053 |
| 6 | - | Phage protein | AAS77054.1 |
| 7 | - | Phage protein | AAS77055 |
| 8 | - | hypothetical protein | none |
| 9 | + | Phage protein | AAS77058 |
| 10 | + | Phage protein | none |
| 11 | + | Phage protein | AAS77059 |
| 12 | + | Phage protein | AAS77061 |
| 13 | + | Phage protein | AAS77062 |
| 14 | - | hypothetical protein | injection stop signal (iss) and putative rho-independent transcription terminator |
| 15 | - | Phage protein | none |
| 16 | - | hypothetical protein | none |
| 17 | - | Phage protein | chee130_1: 15th (66% coverage) |
| 18 | - | Phage protein | none |
| 19 | - | Phage protein | none |
| 20 | - | Phage protein | AAS77069 |
| 21 | - | Phage protein | AAS77071 |
| 22 | - | Phage capsid and scaffold | AAS77072 |
| 23 | - | Phage protein | AAS77074 |
| 24 | - | Phage protein | AAS77075 |
| 25 | - | Phage protein | AAS77076 |
| 26 | - | Phage protein | AAS77077 |
| 27 | - | Phage protein | AAS77079.1 |
| 28 | - | Phage protein | AAS77080 |
| 29 | - | hypothetical protein | none, intergenic |
| 30 | - | Phosphoesterase | AAS77081.1 |
| 31 | - | Serine/threonine protein phosphatase (EC 3.1.3.16) | AAS77082.1 |
| 32 | - | Phage protein | AAS77083 |
| 33 | - | Phage protein | none |
| 34 | - | Thioredoxin, phage-associated | AAS77084 |
| 35 | - | Phage protein | AAS77085 |
| 36 | - | Phage endolysin | AAS77087 |
| 37 | - | Phage holin | AAS77088 |
| 38 | - | ATP-dependent Clp protease proteolytic subunit (EC 3.4.21.92) | AAS77089 |
| 39 | - | Deoxynucleotide monophosphate kinase (EC 2.7.4.13) | AAS77090 |
| 40 | - | Phage protein | AAS77092.1 |
| 41 | - | Phage protein | AAS77094 |
| 42 | - | Phage protein | AAS77095 |
| 43 | - | Phage protein | AAS77096 |
| 44 | - | Phage protein | AAS77097 |
| 45 | - | Phage protein | chee130_1: 42th |
| 46 | - | Phage protein | AAS77099 |
| 47 | - | Phage protein | AAS77100.1 |
| 48 | - | Phage protein | AAS77101.1 |
| 49 | - | Phage protein | AAS77102 |
| 50 | - | Pyruvate formate-lyase (EC 2.3.1.54) | AAS77103 |
| 51 | - | hypothetical protein | none |
| 52 | - | Phage protein | none |
| 53 | - | Phage protein | AAS77105 |
| 54 | - | Phage protein | AAS77202 |
| 55 | - | Phage protein | AAS77106 |
| 56 | - | Phage protein | AAS77108 |
| 57 | - | Phage protein | AAS77109 |
| 58 | - | hypothetical protein | none |
| 59 | - | Phage protein | chee130_1: 56th |
| 60 | - | hypothetical protein | chee130_1: 60th |
| 61 | - | hypothetical protein | chee130_1: 61th |
| 62 | - | Phage protein | AAS77111 |
| 63 | - | Phage protein | AAS77113.1 |
| 64 | - | hypothetical protein | AAS77114 |
| 65 | - | Phage protein | none |
| 66 | - | Phage protein | none |
| 67 | - | Phage protein | AAS77117.1 |
| 68 | - | Phage protein | AAS77118 |
| 69 | - | Phage protein | AAS77119 |
| 70 | - | Phage protein | AAS77120 |
| 71 | - | hypothetical protein | chee130_1: 71th (40% coverage) |
| 72 | + | hypothetical protein | none |
| 73 | + | Phage protein | AAS77122 |
| 74 | - | Phage protein | AAS77123 |
| 75 | - | Phage protein | AAS77124 |
| 76 | - | Phage protein | AAS77126 |
| 77 | - | hypothetical protein | rnaX* |
| 78 | - | Phage recombination related exonuclease (EC 3.1.11.-) | chee130_1: 79th |
| 79 | - | Phage protein | AAS77128.1 |
| 80 | - | Phage protein | AAS77129.1 |
| 81 | - | Phage protein | AAS77130.1 |
| 82 | - | Phage tail fiber protein | AAS77131.1 |
| 83 | - | Phage protein | AAS77132 |
| 84 | - | Metallopeptidase, phage-associated | AAS77133.1 |
| 85 | - | Phage protein | AAS77134.1 |
| 86 | - | Phage tail length tape-measure protein | chee130_1: 87th (partial coverage) |
| 87 | - | Phage protein | AAS77135.1 |
| 88 | - | Phage protein | AAS77136 |
| 89 | - | Phage ribonuclease H (EC 3.1.26.4) | AAS77137.1 |
| 90 | - | hypothetical protein | none |
| 91 | - | Thymidylate synthase (EC 2.1.1.45) | AAS77138 |
| 92 | - | Dihydrofolate reductase, phage-associated | AAS77139 |
| 93 | - | Ribonucleotide reductase of class Ia (aerobic), beta subunit (EC 1.17.4.1) | AAS77140.1 |
| 94 | - | Ribonucleotide reductase of class Ia (aerobic), alpha subunit (EC 1.17.4.1) | AAS77142.1 |
| 95 | - | Phage protein | AAS77143 |
| 96 | - | Phosphate starvation-inducible protein PhoH, predicted ATPase | AAS77144.1 |
| 97 | + | Ribonucleotide reductase of class III (anaerobic), large subunit (EC 1.17.4.2) | AAS77145.1 |
| 98 | + | Phage protein | chee130_1: 102th |
| 99 | + | Phage protein | AAS77147.1 |
| 100 | + | NAD-dependent protein deacetylase of SIR2 family | AAS77148 |
| 101 | + | Phage protein | AAS77150 |
| 102 | + | Phage protein | AAS77151.1 |
| 103 | + | Phage protein | AAS77152 |
| 104 | + | Phage protein | AAS77153 |
| 105 | + | Phage DNA primase C | AAS77154.1 |
| 106 | + | Phage protein | AAS77156 |
| 107 | + | Phage protein | AAS77157.1 |
| 108 | + | Phage protein | AAS77158 |
| 109 | + | Phage protein | AAS77159.1 |
| 110 | + | Phage protein | AAS77160.1 |
| 111 | + | Phage protein | AAS77161 |
| 112 | + | hypothetical protein | none |
| 113 | + | DNA ligase, phage-associated | AAS77162.1 |
| 114 | + | DNA ligase, phage-associated | AAS77163.1 |
| 115 | + | Phage protein | AAS77164.1 |
| 116 | + | Phage protein | AAS77165.1 |
| 117 | + | DNA primase/helicase, phage-associated | AAS77167.1 |
| 118 | + | DNA polymerase I (EC 2.7.7.7), phage-associated | AS77168 |
| 119 | + | Phage protein | AAS77169 |
| 120 | + | DNA helicase, phage-associated | AAS77170.1 |
| 121 | + | Phage protein | AAS77172 |
| 122 | + | Phage-associated recombinase | AAS77173 |
| 123 | + | Phage recombination related exonuclease (EC 3.1.11.-) | AAS77174 |
| 124 | + | Phage protein | AAS77175 |
| 125 | + | Phage ribonuclease H (EC 3.1.26.4) | AAS77176 |
| 126 | + | Deoxyuridine 5'-triphosphate nucleotidohydrolase (EC 3.6.1.23) | AAS77177.1 |
| 127 | + | Phage protein | none |
| 128 | - | Phage tail fibers | none |
| 129 | - | Phage tail fibers | chee130_1: 134, 6% coverage |
| 130 | - | Phage protein | chee130_1: 135th first half |
| 131 | - | Phage tail fibers | chee130_1: 135th second half |
| 132 | - | Phage tail length tape-measure protein | AAQ92754.1 |
| 133 | - | Phage protein | none |
| 134 | - | Phage tail length tape-measure protein | AAQ92756.2 |
| 135 | - | Phage protein | AAS77181 |
| 136 | - | Phage protein | AAS77182.1 |
| 137 | - | Phage tail fibers | AAS77183.1 |
| 138 | - | Phage major tail protein | AAS77184 |
| 139 | - | Phage protein | AAS77185 |
| 140 | - | Phage protein | AAS77186 |
| 141 | - | Phage protein | AAS77187 |
| 142 | - | Phage capsid and scaffold | AAS77188 |
| 143 | - | Phage capsid and scaffold | AAS77189 |
| 144 | - | Phage tail fibers | AAS77190 |
| 145 | - | Phage portal (connector) protein | AAS77191 |
| 146 | - | Phage protein | AAS77192 |
| 147 | - | Phage terminase, large subunit | AAS77194 |
| 148 | - | Phage protein | AAS77195 |
| 149 | - | Phage-associated receptor-binding protein | none |
| 150 | + | Phage protein | none |
| 151 | + | Phage protein | AAS77199 |
| 152 | + | Phage protein | AAS77200 |
| 153* | - | degrades 5'-deoxyribonucleotides to their corresponding deoxyribonucleosides | AAS77048 |
| 154* | - | Phage protein | AAS77049 |
| 155* | - | hypothetical protein | none |
| 156* | - | Probable A1 protein | AAS77051 |
| 157* | - | A2 protein | AAS77053 |
| 158* | - | Phage protein | AAS77054.1 |
| 159* | - | Phage protein | AAS77055 |
| 160* | - | hypothetical protein | none |
| 161* | + | Phage protein | AAS77058 |
| 162* | + | Phage protein | none |
| 163* | + | Phage protein | AAS77059 |
| 164* | + | Phage protein | AAS77061 |
| 165* | + | Phage protein | AAS77062 |
| 166* | - | hypothetical protein | injection stop signal (iss) and putative rho-independent transcription terminator |

* right inverted repeat, identical genes to 1-14

**Supplementary Table 3. Comparative list of ORFs in T5-like bacteriophages representing the chee130_1-like subgroup.**

| **ORF** | **Strand** | **Predicted function** | **Corresponding ORF in T5 or chee24** |
| --- | --- | --- | --- |
| 1 | - | degrades 5'-deoxyribonucleotides to their corresponding deoxyribonucleosides | AAS77048.1 |
| 2 | - | Phage protein | none |
| 3 | - | Probable A1 protein | AAS77051.1 |
| 4 | - | A2 protein | none |
| 5 | - | Phage protein | none |
| 6 | - | hypothetical protein | none |
| 7 | + | Phage protein | none |
| 8 | + | Phage protein | none |
| 9 | + | Phage protein | none |
| 10 | + | hypothetical protein | none |
| 11 | + | Phage protein | none |
| 12 | - | Phage protein | none |
| 13 | - | Phage protein | none |
| 14 | - | Phage protein | none |
| 15 | - | Phage protein | none |
| 16 | - | Phage protein | none |
| 17 | - | Phage protein | none |
| 18 | - | Phage protein | none |
| 19 | - | Phage capsid and scaffold | none |
| 20 | - | Phage protein | chee24: 23th |
| 21 | - | Phage protein | none |
| 22 | - | Phage protein | none |
| 23 | - | Phage protein | none |
| 24 | - | Phage protein | none |
| 25 | - | hypothetical protein | none |
| 26 | - | Phage protein | AAS77079.1 |
| 27 | - | Phage protein | none |
| 28 | - | Phosphoesterase | AAS77081.1 |
| 29 | - | Phage protein | none |
| 30 | - | Serine/threonine protein phosphatase (EC 3.1.3.16) | AAS77082.1 |
| 31 | - | Phage-associated homing endonuclease | none |
| 32 | - | Thioredoxin, phage-associated | chee24: 34th |
| 33 | - | Phage-associated homing endonuclease | none |
| 34 | - | Phage protein | none |
| 35 | - | Phage endolysin | AAS77087.1 |
| 36 | - | Phage holin | AAS77088.1 |
| 37 | - | ATP-dependent Clp protease proteolytic subunit (EC 3.4.21.92) | AAS77089.1 |
| 38 | - | Deoxynucleotide monophosphate kinase (EC 2.7.4.13) | AAS77090.1 |
| 39 | - | Phage protein | AAS77092.1 |
| 40 | - | Phage protein | none |
| 41 | - | Phage protein | none |
| 42 | - | Phage protein | none |
| 43 | - | Phage protein | none |
| 44 | - | Phage protein | chee24: 42th |
| 45 | - | Phage protein | chee24: 43th |
| 46 | - | Phage protein | AAS77100.1 |
| 47 | - | Phage protein | AAS77101.1 |
| 48 | - | Phage protein | none |
| 49 | - | hypothetical protein | none |
| 50 | - | Pyruvate formate-lyase (EC 2.3.1.54) | AAS77103.1 |
| 51 | - | Phage protein | none |
| 52 | - | Phage protein | AAS77202.1 |
| 53 | - | Phage protein* | AAS77106.1 |
| 54 | + | hypothetical protein | none |
| 55 | - | Phage protein | none |
| 56 | - | hypothetical protein | none |
| 57 | - | Phage protein | none |
| 58 | - | Phage protein | chee24: intergenic |
| 59 | - | Phage protein | chee24: 59th |
| 60 | - | hypothetical protein | chee24: 60th |
| 61 | - | hypothetical protein | chee24: 61th |
| 62 | - | Phage protein | none |
| 63 | + | hypothetical protein | AAS77113.1 |
| 64 | - | hypothetical protein | none |
| 65 | - | Phage protein | none |
| 66 | - | Phage protein | AAS77117.1 |
| 67 | - | Phage protein | none |
| 68 | - | Phage protein | none |
| 69 | - | Phage protein | none |
| 70 | - | Phage protein | none |
| 71 | - | Phage antitermination protein Q | chee24: 71th (40% coverage) |
| 72 | - | hypothetical protein | none |
| 73 | - | Phage protein | none |
| 74 | - | Phage protein | none |
| 75 | - | Ribosyl nicotinamide transporter, PnuC-like | none |
| 76 | - | NadR transcriptional regulator / Nicotinamide-nucleotide adenylyltransferase, NadR family (EC 2.7.7.1) / Ribosylnicotinamide kinase (EC 2.7.1.22) | none |
| 77 | - | Phage protein | AAS77134.1 |
| 78 | - | Phage protein | none |
| 79 | - | Phage protein | chee24: 78th |
| 80 | - | Phage protein | AAS77128.1 |
| 81 | - | Phage protein | AAS77129.1 |
| 82 | - | Phage protein | AAS77130.1 |
| 83 | - | Phage tail fiber protein | AAS77131.1 |
| 84 | - | Phage protein | chee24: 83th |
| 85 | - | Metallopeptidase, phage-associated | AAS77133.1 |
| 86 | - | Phage protein | AAS77134.1 |
| 87 | - | Phage tail length tape-measure protein | chee24: 86th (partial coverage) |
| 88 | - | Phage protein | AAS77135.1 |
| 89 | - | Phage protein | none |
| 90 | - | Ribonuclease HI (EC 3.1.26.4) | AAS77137.1 |
| 91 | - | Phage protein | none |
| 92 | - | Phage protein | none |
| 93 | - | Thymidylate synthase (EC 2.1.1.45) | none |
| 94 | - | Dihydrofolate reductase, phage-associated | none |
| 95 | - | Ribonucleotide reductase of class Ia (aerobic), beta subunit (EC 1.17.4.1) | AAS77140.1 |
| 96 | - | Phage-associated homing endonuclease | none |
| 97 | - | Ribonucleotide reductase of class Ia (aerobic), alpha subunit (EC 1.17.4.1) | AAS77142.1 |
| 98 | - | Phage protein | chee24: 62th (76% coverage) |
| 99 | - | Phosphate starvation-inducible protein PhoH, predicted ATPase | AAS77144.1 |
| 100 | + | Ribonucleotide reductase of class III (anaerobic), large subunit (EC 1.17.4.2) | AAS77145.1 |
| 101 | + | unknown | none |
| 102 | + | Phage protein | chee24: 98th |
| 103 | + | Phage protein | AAS77147.1 |
| 104 | + | hypothetical protein | none |
| 105 | + | NAD-dependent protein deacetylase of SIR2 family | none |
| 106 | + | Phage protein | none |
| 107 | + | Phage protein | none |
| 108 | + | Phage protein | AAS77151.1 |
| 109 | + | Phage protein | chee24: 103th |
| 110 | + | Phage protein | none |
| 111 | + | Phage DNA primase C | AAS77154.1 |
| 112 | + | Phage protein | AAS77157.1 |
| 113 | + | hypothetical protein | none |
| 114 | + | Phage protein | AAS77159.1 |
| 115 | + | Phage protein | AAS77160.1 |
| 116 | + | Phage protein | AAS77161.1 |
| 117 | + | hypothetical protein | none |
| 118 | + | hypothetical protein | none |
| 119 | + | DNA ligase, phage-associated | AAS77162.1 |
| 120 | + | DNA ligase, phage-associated | AAS77163.1 |
| 121 | + | Phage protein | AAS77164.1 |
| 122 | + | Phage protein | AAS77165.1 |
| 123 | + | DNA primase (EC 2.7.7.-) / DNA helicase (EC 3.6.1.-), phage-associated | AAS77167.1 |
| 124 | + | DNA polymerase I (EC 2.7.7.7), phage-associated | AAS77172.1 |
| 125 | + | DNA helicase, phage-associated | AAS77170.1 |
| 126 | + | Phage-associated homing endonuclease | none |
| 127 | + | Phage protein | AAS77172.1 |
| 128 | + | Phage-associated recombinase | AAS77173.1 |
| 129 | + | Phage recombination related exonuclease (EC 3.1.11.-) | AAS77174.1 |
| 130 | + | hypothetical protein | AAS77175.1 |
| 131 | + | Phage ribonuclease H (EC 3.1.26.4) | AAS77176.1 |
| 132 | + | Deoxyuridine 5'-triphosphate nucleotidohydrolase (EC 3.6.1.23) | AAS77177.1 |
| 133 | + | Phage protein | none |
| 134 | - | Phage tail fibers | chee24: 145, (7% coverage) |
| 135 | - | Phage protein | chee24: 146 |
| 136 | + | Phage tail fibers | chee24: 147 |
| 137 | + | Phage tail length tape-measure protein | AAQ92754.1 |
| 138 | + | Phage protein | AAQ92755.1 |
| 139 | + | Phage tail length tape-measure protein | AAQ92756.2 |
| 140 | + | Phage protein | AAS77181 |
| 141 | + | Phage protein | AAS77182.1 |
| 142 | + | Phage tail fibers | AAS77183.1 |
| 143 | + | Phage major tail protein | AAS77184.1 |
| 144 | - | hypothetical protein | AAS77185.1 |
| 145 | - | Phage protein | AAS77186.1 |
| 146 | - | Phage protein | none |
| 147 | - | Phage major capsid protein | AAS77188.1 |
| 148 | - | Phage capsid and scaffold | AAS77189.1 |
| 149 | - | Phage neck whiskers | AAS77190.1 |
| 150 | - | Phage portal (connector) protein | AAS77191.1 |
| 151 | - | Phage protein | AAS77192.1 |
| 152 | - | Phage terminase, large subunit | AAS77193.1 |
| 153 | - | Phage protein | AAS77195.1 |
| 154 | - | Phage-associated receptor-binding protein | none |
| 155 | + | Phage protein | none |
| 156* | - | degrades 5'-deoxyribonucleotides to their corresponding deoxyribonucleosides | AAS77048.1 |
| 157* | - | Phage protein | none |
| 158* | - | Probable A1 protein | AAS77051.1 |
| 159* | - | A2 protein | none |
| 160* | - | Phage protein | none |
| 161* | - | hypothetical protein | none |
| 162* | + | Phage protein | none |
| 163* | + | Phage protein | none |
| 164* | + | Phage protein | none |
| 165* | + | hypothetical protein | none |
| 166* | + | Phage protein | none |
| 167* | - | Phage protein | none |

* right inverted repeat, identical genes to 1-12
